# Molecular biology of carcinoid heart disease: Seeking therapeutic targets in the era of targeted therapy

**DOI:** 10.1097/MD.0000000000036043

**Published:** 2023-11-24

**Authors:** Sajjad Ahmed Khan, Dikshya Devkota, Rahul Kumar Chaudhary, Anish Luitel, Surya Bahadur Parajuli, Madhab Bista, Asmita Rayamajhi

**Affiliations:** a Birat Medical College Teaching Hospital, Morang, Nepal; b Department of Anesthesiology and Critical Care, Birat Medical College Teaching Hospital, Morang, Nepal; c Department of Community Medicine, Birat Medical College Teaching Hospital, Morang, Nepal; d Department of Cardiovascular Medicine, Birat Medical College Teaching Hospital, Morang, Nepal; e Department Radiation Oncology, Birat Medical College Teaching Hospital, Morang, Nepal.

**Keywords:** carcinoid syndrome, cardiovascular manifestations, heart

## Abstract

Carcinoid tumors (CT) are among the least studied tumors. It is a relatively rare and slow-growing tumor with good survival in its primary form. However, metastatic carcinoid tumor comes up with many different complications contributing to increased morbidity and mortality. Metastatic form of the disease has a wide spectrum of clinical manifestations and multisystem involvement including cardiovascular manifestations, neurological manifestations, and psychiatric manifestations and so on. In this review, we are centered towards the cardiovascular manifestations of the disease, which, by far, is one of the commonest causes of death in these patients. Being a neuroendocrine tumor, cardiovascular manifestations are mainly because of biologically active substances, produced by the tumor cells, remodeling the heart. Development of targeted therapies against carcinoid heart diseases is currently limited by lack of understanding of pathophysiology of the disease processes. In this review, we aim to figure out the spectrum of carcinoid heart diseases, along with the molecular biology of the changes going on, which, in turn, will not only pave a way to the development of targeted therapies but will also extend opportunities for molecular screening of the tumor and diagnosis at an early stage, thereby, reducing the morbidity and mortality.

## 1. Introduction

Based on molecular background, carcinoid tumor has been divided into foregut, midgut, and hindgut tumors.^[1]^ Foregut carcinoid tumors (CTs) commonly originate in the lungs, bronchi, or stomach; midgut CTs in the small intestine, appendix, or proximal large bowel; and hindgut CTs in the distal colon or rectum.^[2]^ Talking about the distribution of CTs, small intestine is the most common site of carcinoids (45%), followed by the rectum (20%), appendix (17%), colon (11%), and stomach (7%). Less common anatomic sites have been reported in the literature, including carcinoids of the breast, larynx, thymus, and gallbladder.^[3]^ As for primary tumor location, tumors arising from the small intestine account for 31% to 80% of all carcinoids and In a 4 series of carcinoid patients with heart disease, however, small-intestine primaries accounted for 87% to 100% of cases.^[4]^ Carcinoid tumors involve the heart in up to 60% of patients and cardiac involvement is associated with a poor long-term prognosis with the estimated 3-year survival rate of 31% is half that of patients without cardiac involvement.^[5]^ Carcinoid heart disease (CHD) is a rare and potentially lethal manifestation of an advanced carcinoid (neuroendocrine) tumor. The development of CHD is a fibrotic complication of neuroendocrine neoplasms which is associated with a poor prognosis.^[6]^ Clinical presentations of CHD vary depending upon the region of heart being involved; most typical is right-sided heart failure secondary to diseased tricuspid and pulmonary valves.^[5]^ Management of CHD is the most challenging part. So far now, we do not have any approved antiproliferative agent to be used in carcinoids due to inadequacy of knowledge on tumor biology.^[7]^ Monoclonal antibody therapy and other chemotherapeutic agents like Trastuzumab in combination with IL-12, new somatostatin analogue like lanreotide is under investigations.^[5]^ So far there use in carcinoid heart disease is concerned, they have been used as off-label drugs in some centers with questionable efficacy; however no standard consensus exist regarding their use in these group of patients.

There are different biogenic amines which are of interest with regard to the progression of the disease process and subsequent development of targeted therapies. Among those amines, it has been found that serotonin is the key neurotransmitter involved. Carcinoid tumors secrete serotonin which comes in contact with liver via portal circulation and in the liver this biogenic amine gets degraded by deaminases present in the liver and no cardiac manifestation will be seen if the disease remains localized to its primary site.^[8]^ However, if metastasis to liver has occurred, these biogenic amines will escape the hepatic metabolism and get into systemic venous circulation affecting the right-sided heart; from the right-heart serotonin will get access to the lungs via pulmonary circulation and in the lungs it again get deactivated by the deaminases present in the lungs sparing the left heart. Hence, hepatic metastasis of the disease will only affect the right-heart and for left-heart to be affected, tumor should get spread to lungs, as well.^[9]^ Targeting serotonin, its receptors present in the heart and modulating the molecular pathways induced by serotonin in the heart to bring about the spectrum of changes seen in carcinoid heart disease, will definitively be a very promising way to halt the progression of the disease. In this review, we attempt to explore the tumor biology, which will allow pave a way for molecular biologist and clinician to work on targeted therapies specific to the biogenic amines and molecular pathways involved.

## 2. Methodology

### 2.1. Literature search

The following databases were reviewed for published studies prior to 18th of September, 2022: PubMed, Cochrane Library, and Directory of Open Access Journals. Boolean logic was used for conducting database search and Boolean search operators “AND” and “OR” were used to link search terms. Also, parenthesis, double inverted comas were used while searching for phrases. The following search strategy was adopted: “Carcinoid syndrome” AND (“Cardiovascular manifestations” OR Heart). Keeping in mind the eligibility criteria, title, abstract, and full text was screened. Two authors (SAK and DD) screened, retrieved, and excluded reports. Additional investigators were consulted if uncertainty arose during the review process.

### 2.2. Eligibility criteria

We included any study, published in English language, dealing with the molecular biology of carcinoid heart disease secondary to gastrointestinal CT. We excluded the articles published in languages other than English and we also excluded articles dealing with carcinoid syndromes of location other than gastrointestinal system.

### 2.3. Data extraction

Data extraction was done manually from eligible studies by the research investigators.

### 2.4. Outcome measures

Our outcome was to explore the tumor biology of carcinoids, thus providing insight into screening measures and potential interventional strategies. Needful information was taken selectively from eligible studies (Table 1).

**Table 1:** Characteristics of studies included in this narrative review.

SN	Primary author	Type of publication	Country	Journal	Information extracted
1	Kejjel oberg	Review article	Sweden	Current opinion in oncology	Types of carcinoid tumor: foregut, midgut, hindgut
2	Laskaratos	Review article	United Kingdom	Current oncology reports	Carcinoid heart disease is a fibrotic process with poor prognosis
3	Ram P	Review article	United States	Texas Heart Institute Journal	Valvular heart diseases, arrhythmias, coronary artery disease, myocardial involvement
4	Pavel M	Review article	Germany	Seminars in oncology	No established systemic therapeutic option for carcinoids
5	Pinchot SN	Review article	United States	The Oncologist	Enterochromaffin cells disseminated throughout GIT
6	Jin C	Review article	United States	Cardiology Journal	Hepatic monooxidase inactivate 5-HT
7	Hutcheson JD	Review article	United States	Pharmacology and Therapeutics	5HT found in blood platelets and enterochromaffin cells of the gut and neurons
8	Roth BL	Review article	United States	Pharmacology and Therapeutics	Functions of serotonin: sleep, mood, appetite, anxiety, aggression, perception, pain, and cognition
9	Pytliak M	Review article	Slovak Republic	Physiological Research	Gq mediated action of 5HT, homology between 5HT2 receptors subtypes
10	Gustafsson BI	Animal study	Norway	Circulation	Valvulopathies in rat similar to carcinoids in human after long-term 5HT administration
11	Elangbam CS	Animal study	United States	Experimental and Toxicological Pathology	Increased 5HT_2B_ receptor expression in carcinoids
12	Liu AC	Review article	Canada	The American Journal of Pathology	three layers of valves - the fibrosa, the spongiosa, and the ventricularis with ICs and endothelial cells
13	Chester AH	Review article	United Kingdom	The Royal society publishing	ICs form a network within ECM within the body of the cusp, maintaining the integrity of the cusps
14	Mongkoldhumrongkul N	Review Article	United Kingdom	Current Vascular Pharmacology	Endothelial cells exposed to blood flow, ICs secretes connective tissues
15	Lester W	Experimental study	Canada	Laboratory Investigation	ICs functions like myofibroblasts
16	Sappino AP	Review Article	Switzerland	Laboratory Investigation	Collagens, elastin, proteoglycan synthesize by ICs
17	Taylor PM	Experimental Study	United Kingdom	The Journal of Heart Valve Disease	Myofibroblast type and secretory type of ICs
18	Arora PD	Observational Study	Canada	Journal of cellular physiology	Myofibroblasts type of ICs
19	Janin A	Observational study	France	Clinical and Experimental Rheumatology	Enzyme prolyl 4-hydroxylase in secretory ICs
20	Konttinen YT	Experimental Study	Finland	Journal of Rheumatology	Collagen synthesis by secretory ICs
21	Latif N	Experimental study	United Kingdom	Cell Biochemistry and Biophysics	ICs communicates with each other and ECM
22	Rutkovskiy A	Review Article	Norway	Journal of American Heart Association	Osteoblastic differentiation of ICs
23	Goldberg E	Review Article	United States	Cardiovascular Research	5HT_2B_ receptor in Heart
24	Nebigil CG	Experimental study	France	Circulation	Decreased ventricular mass and absence of trabeculae and intercalated disc in 5HT deficient state
25	Nebigil CG	Experimental study	France	Proceedings of the National Academy of Sciences of the United States of America	ICs helps to maintain ventricular mass
26	Jian B	Experimental study	United states	The American Journal of Pathology	Hyperplastic valvular and endocardial lesions with increased extracellular matrix seen with excess 5HT
27	Buskohl	Experimental study	United States	PLoS one	5HT leads to upregulation of TGF- β via Gq pathway
28	Fitzgerald	Observational study	United States	Molecular Pharmacology	5HT pathways on ICs
29	Jian B	Experimental study	United States	The Annals of Thoracic Surgery	TGF- β leads to pathologic ECM remodeling
30	Lacelloti P	Experimental study	Belgium	International Journal of Cardiology	Exogenous 5HT administration has shown the evidence of heart valve diseases in study conducted in adult mammals
31	Ni W	Review article	United States	Clinical and Experimental Pharmacology and Physiology	SERT dependent serotonin entry into the cells
32	Rajamanickam J	Observational study	United States	Biochemical Journal	Akt phosphorylation leading to down regulation of SERT
33	Scruggs SM	Experimental study	United States	Journal of Veterinary Cardiology	SERT downregulation in advanced heart valve disease
34	Jaffre F	Experimental study	France	Circulation Research	Angiotensin receptor 1 (AT-1) and 5HT_2B_ receptors have been shown to work in collaboration with each other
35	O Brein KD	Observational study	United States	Circulation	AT-1 is upregulated in αSMA-expressing VICs
36	Veenendaal LM	Review article	Netherlands	Word Journal of Surgical Oncology	Right heart involvement when 5HT escapes hepatic metabolism
37	Yuan SM	Review article	China	Brazilian Journal of Cardiovascular Surgery	Morbidity and mortality with right heart failure
38	Nielsen MS	Case report	United Kingdom	Anaesthesia	Tricuspid regurgitation, being typical of carcinoid heart disease
39	Fox DJ	Review article	United Kingdom	Heart	Endocardial plaques of fibrous tissue involving the valve and the subvalvular components lead to tricuspid regurgitation
40	Hassan SA	Review article	United States	Heart	Half of patients with carcinoid syndrome can have carcinoid heart disease
41	Sanchez-Nadales	Case report	United States	Journal of Medical Cases	Right heart failure in carcinoids
42	Schweizer W	Case report	United States	Circulation	Left sided valvular pathology is due to either extensive liver metastases, bronchial carcinoid, a patent foramen ovale
43	Strickman NE	Review article	United States	Current Problems in Cardiology	Detoxification system at Lungs get overwhelmed leading to left heart carcinoids.
44	Kulke MH	Review article	United States	New England Journal of Medicine	patients with heart disease have 2- to 4-fold higher levels of serotonin in their serum
45	Bourgault	Case report	Canada	Canadian Journal of Cardiology	5-HIAA in urine of patients with carcinoids
46	Chu A	Experimental study	United States	Circulation Research	Biphasic vasomotor response of 5HT
47	McFadden	Comparative study	United Kingdom	New England Journal of Medicine	Carcinoid-induced coronary vasospasm in patients who have non occlusive coronary disease
48	Ardlie NG	Review article	United States	Thrombosis Research	5HT, being a platelet activator, can cause coronary spasm via direct entry through patent foramen ovale
49	Rupp AB	Case report	United States	Case Reports in Cardiology	Ventricular tachycardia in carcinoids
50	Tappin S	Case report	United Kingdom	Journal Of Small Animal practice	Carcinoid syndrome causing ventricular tachycardia in a canine subject
51	Bhattacharyya S	Observational study	United Kingdom	Circulation Cardiovascular Imaging	Incidence of cardiac metastasis in carcinoids is 3.8%
52	Pandya UH	Observational study	United States	Journal of the American College of Cardiology	Well circumscribed, homogenous mass and non-infiltrative on echocardiogram was seen in cases of ventricular metastasis
53	Bonsen LR	Review article	Netherlands	Nuclear Medicine Communications	Extent of metastases to heart reflects the degree of tumor burden
54	Noordzij W	Observational study	Netherlands	PLoS One	Cardiac carcinoid metastases did not correlate with other classic valvular lesions of the carcinoid heart diseases
55	Wang TS	Case report	United States	Journal of the American College of Cardiology	Pericardial metastasis of carcinoids
56	Rich L.L.	Case report	United States	The American Journal of Medicine	Chronic pericarditis, fibrosis, acute pericarditis, secondary to carcinoid tumor
57	Johnston S.D.	Case report	United Kingdom	Heart	Constrictive carcinoid pericarditis without valve involvement
58	Lepska L	Case report	Poland	Kardiologia Polska	Massive pericardial effusion in carcinoids
59	Naraev BG	Review article	United States	Pancreas	5HT leads to increased peristalsis, which results in reduced absorption of water and electrolytes, leading to diarrhea
60	Rastogi V	Review article	United States	Clinical Medicine and Research	Flushing is because of vasodilator effect of 5HT on cutaneous blood vessels
61	Ito T	Review article	Japan	Current Opinion in Endocrinology Diabetes and Obesity	Varying clinical presentations and often refractory to treatment
62	Ito T	Review article	Japan	Expert Opinion on Pharmacotherapy	Telotristat, peptide radioreceptor therapy, radiolabeled somatostatin analogues, ^131^MIBG

5HT = 5-hydroxytryptamine, GIT = gastrointestinal system, ^131^MIBG = iodine meta-iodobenzylguanidine.

### 2.5. Quality assessment

The quality of included studies was assessed using appropriate tools, such as the Newcastle-Ottawa Scale for non-randomized studies and the Cochrane risk of bias tool for randomized controlled trials. Studies with a high risk of bias were carefully considered during the interpretation of results.

### 2.6. Overview of carcinoid tumor

Carcinoid tumors are rare, slow-growing neuroendocrine tumors arising from the enterochromaffin cells disseminated throughout the gastrointestinal tract.^[1]^ The term “carcinoid” was coined in 1907 by the German pathologist, Siegfried Oberndorfer, who discovered and named the tumor “karzinoid” or “carcinoma-like,” to describe the unique behavior of a benign tumor despite a malignant microscopic appearance.^[6]^ Clinical features produced by the CT are mainly due to release of biologically active substances particularly serotonin by the enterochromaffin cells. A primary CT cannot produce cardiovascular manifestations because biologically active amines produced by the tumor cells is poured into portal circulation, from where, it reaches the liver and get degraded by hepatic monoamine oxidases preventing accessibility to systemic circulation.^[6]^

### 2.7. Overview of carcinoid syndrome

Carcinoid syndrome is an overarching term including a constellation of symptoms caused by the release of humoral factors such as polypeptides, prostaglandins, and biogenic amines by the tumor cells. It arises when biologically active substances produced by the tumor cells reach to systemic circulation escaping the first pass metabolism at liver. Midgut carcinoids are more frequently associated with carcinoid syndrome compared to foregut and hindgut carcinoids.^[6]^

### 2.8. Serotonin and the heart valve serotonin and its receptors

Serotonin or 5-hydroxytryptamine (5-HT) is a neurotransmitter synthesized following hydroxylation and decarboxylation of tryptophan. High concentrations of 5-HT are found in blood platelets and enterochromaffin cells of the gut; lesser amounts are found around neurons located along the raphé nuclei of the brainstem.^[5]^ In the brain, 5-HT has been found to affect sleep, mood, appetite, anxiety, aggression, perception, pain, and cognition.^[7]^ Talking about the receptors of serotonin, there are altogether of 7 different types of 5-HT receptors acting via G-coupled receptor mediated action except 5-HT_3_ receptor which is a ligand gated Na+/K+ channel.^[10]^ The 5-HT_2_ family consists of 3 GPCRs: 5-HT_2A_, 5-HT_2B_, and 5-HT_2C_, with 40–50% of homology amongst the receptor subtypes, which is of significant importance, as drugs targeted to 1 subtype act on other, as well due to such a high degree of similarity.^[10]^ Long term administration of serotonin in rats has shown valvulopathies similar to that of human carcinoid heart diseases.^[11]^ Increased 5-HT_2B_ receptor expression with decreased expression of 5-HT transporter in both mitral and aortic valves has been attributed to the valvular changes.^[12]^

### 2.9. The heart valves

To understand the effect of these biogenic amines on the heart valve, it is important to review the normal structural, functional and biochemical aspects of these valves. There are total of 4 valves in the heart: mitral valve, tricuspid valve, aortic valve, and pulmonic valve, containing 3 layers – the fibrosa, the spongiosa, and the ventricularis.^[13]^ There are principally 2 types of cells found in cusp tissue: the endothelial cells (ECs) that cover the surface of the cusps and the interstitial cells (ICs) that form a network within the extracellular matrix (ECM) within the body of the cusp, maintaining the integrity of the cusps.^[14]^

### 2.10. The endothelial cells

The ECs present in the inflow and outflow surface of the valve are different owing to difference in pressure they are exposed to, ensuring that the valve function are in optimal manner.^[14,15]^ It has been postulated that ECs also stimulates ICs for secretion and synthesis of glycosaminoglycan, proteoglycan, glycoprotein, and other connective tissue matrix, thus contributing to calcification and degeneration of the valve.^[15]^

### 2.11. The interstitial cells

Valve ICs functions like myofibroblasts but are not phenotypically similar to fibroblasts having many unique characteritics.^[16]^ These cells synthesize matrix components, such as collagen, elastin, proteoglycans, and glycoproteins; growth factors, cytokines, and chemokines; as well as matrix remodeling enzymes, the matrix metalloproteinases, and their tissue inhibitors.^[14,17]^ Phenotypically, 2 different phenotypes of ICs are documented via electron microscopy and immunohistochemistry evaluation.^[14]^ The myofibroblasts type of ICs is concerned with smooth muscle α-actin and is thought to be proliferative, migratory, and capable of remodeling the ECM.^[18,19]^ The second phenotype is mainly concerned with secretory and synthetic activity which expresses the enzyme prolyl 4-hydroxylase for stabilization of the collagen triple helix, which indicates that the cells are actively synthesizing collagen.^[20,21]^ Another, unique feature of the ICs is the fact that they communicate with each other and ECM to maintain the structural integrity of the valve.^[22]^ ICs can also undergo pathological differentiation to osteoblasts.^[14]^ Practically any ICs can differentiate into osteoblasts when stimulated with appropriate stimuli which explains a large versatility of ectopic calcification that may occur after tissue injury in most parts of the body, and the VICs are no exception.^[23]^ Thus, with adequate stimulus for osteoblast differentiation of ICs, calcification associated valvulopathies can occur.

### 2.12. Role of serotonin in remodeling the heart valve

The 5HT_2B_ receptor is expressed throughout cardiac tissues and is believed to play an important role in development of the heart.^[24]^ In experiment conducted in mice designed by homologous recombination to lack the 5HT_2B_ receptor, decreased ventricular mass and absence of trabeculae and intercalated disc was noted.^[25,26]^ Clinical disorders associated with increased serotonin 5-HT levels, such as carcinoid syndrome, and the use of serotonin agonists, such as fenfluoramine have been associated with a valvulopathy characterized by hyperplastic valvular and endocardial lesions with increased extracellular matrix.^[27]^ Studies done in birds suggest that 5HT may induce these remodeling effects through a TGF-β-dependent mechanism.^[28]^

5HT_2_ receptors, a group of serotonin receptors (5HT_2A_, 5HT_2B_, 5HT_2C_) involved in heart diseases and are coupled to Gα_q_ pathways,^[29,30]^ 5-HT acting on its receptor on ICs activates phospholipase C via Gq pathway which in turn, leads to up regulation of TGF-β.^[30]^ The fibrogenic cytokine TGF-β is known for pathologic ECM remodeling in many different organs and Heart is no exception.^[31]^ In the heart, TGF-β activates ICs by promoting differentiation, migration, matrix condensation, angiogenesis and NADPH oxidase activity generating reactive oxygen species which encourage synthesis of TGF-β, initiating a positive feedback loop of ICs activation.^[24]^ Exogenous 5HT administration has shown the evidence of heart valve diseases (HVDs) in study conducted in adult mammals.^[11,32]^ Not surprisingly, upregulation of 5HT_2_ receptor mRNA has been repeatedly observed in diseased heart valve leaflets, a process that may increase the sensitivity of these tissues to 5HT by increasing the number of available receptors.^[24]^

Together with receptor mediated action, 5HT directly enter the cell via a sodium-dependent transporter protein, serotonin transporter (SERT), which downgrade its function at plasma membrane receptors by reducing the amount of circulating 5HT.^[33]^ Via increased Akt phosphorylation, downregulation of SERT occurs in advanced stage of HVDs.^[34,35]^ Thus, it is hypothesized that downregulation of SERT may be a contributing factor in HVDs, apart from, direct action of 5-HT on ICs.

Another interesting fact is association of 5HT signaling in ICs with renin–angiotensin system, particularly through the peptide hormone angiotensin II.^[24]^ Recently, the angiotensin receptor 1 (AT-1) and 5HT_2B_ receptors have been shown to work in collaboration with each other in a way that both are required for the activity of either 1.^[36]^ Angiotensin II and its immediate source, angiotensin converting enzyme, are upregulated and colocalize with lesions in diseased aortic valves.^[24]^ AT-1 is generally absent from healthy valve tissues but is upregulated in αSMA-expressing VICs, implying that AT-1 expression may be a characteristic of pathologically differentiated fibroblasts.^[24,37]^

### 2.13. Spectrum of carcinoid heart disease right-sided carcinoid heart disease

For the right heart to get involved, metastases to liver is needed and the biogenic amines should be poured directly to the systemic circulation or liver function should be deranged to the degree where it cannot detoxify the amines released by the tumor cell.^[38]^ Once the carcinoid syndrome has developed, approximately 50% of these patients develop carcinoid heart disease which typically causes abnormalities of the right side of the heart.^[39]^ It is an important cause of intrinsic right heart valve disorders with significant morbidity and mortality secondary to right heart failure.^[40]^ Following are the clinical manifestations along with the pathophysiology for right heart carcinoid diseases:

### 2.14. Tricuspid valve disease

Tricuspid regurgitation (TR) is the typical valvular lesion associated with carcinoid heart disease.^[41]^ The characteristic pathological findings are endocardial plaques of fibrous tissue involving the valve and the subvalvular components including valve leaflets, but also the subvalvular apparatus including the tendinous chords and papillary muscles, resulting mainly TR.^[39]^ TR was almost universal at 92% to 100%, followed by tricuspid stenosis (38–44%).^[40]^

### 2.15. Pulmonic valve disease

Most commonly, pulmonic valve involvement presents as mixed pulmonic regurgitation (31–38%) and pulmonic stenosis (25–31%).^[40]^ Pathophysiology of pulmonic valve diseases secondary to carcinoid syndrome is similar to that of tricuspid valve diseases, that is, fibrous tissue in the plaques results in distortion of the valves leading to either stenosis, regurgitation, or both.^[40]^

### 2.16. Right heart failure

More than half of patients with carcinoid syndrome can have carcinoid heart disease, presenting with valvular involvement and symptoms of heart failure (mostly edema, ascites and shortness of breath) typically involving right heart in 50% cases.^[42,43]^ Valvular pathology along with the fibrous reaction going on the cardiac chambers and endocardium results in the heart failure.^[40]^

### 2.17. Left-sided carcinoid heart disease

In the 5% to 10% of cases with left sided valvular pathology, one should suspect either extensive liver metastases, bronchial carcinoid, a patent foramen ovale, or metastases to Lungs overwhelming the detoxification process going on in the lungs.^[44,45]^ Not surprisingly, it has been found that Patients with heart disease have 2- to 4-fold higher levels of serotonin in their serum and 5-HIAA in their urine than other patients with the carcinoid syndrome.^[46,47]^

### 2.18. Coronary artery involvement

Experimental studies done in dogs have shown that 5HT effects a biphasic vasomotor response characterized by an initial vasodilation that is mediated primarily through a direct endothelium-dependent 5HT_1_ receptor mediated action followed by a delayed vasoconstriction that is probably mediated via a direct 5HT_2_ receptor effect on the vascular smooth muscle and is attenuated by the normal endothelium.^[48]^ Thus, in diseased endothelium (as in atherosclerotic disorders), where there is predominance of vasoconstriction-provoking 5-HT_2_ receptors and the loss of 5-HT_1_ receptors that mediate vasodilation, 5HT can cause vasoconstriction.^[5]^ This response explains the finding of carcinoid-induced coronary vasospasm in patients who have non occlusive coronary disease.^[5,49]^

In patients with right to left shunt (patent Foramen ovale), 5HT can directly get access to coronary circulation and contributes thrombosis, being a known platelet activator.^[50]^

### 2.19. Cardiac arrhythmias

Proposed mechanism of cardiac arrhythmias is multiple bioactive amines release by the tumor cells, leading to cardiac excitation and tachyarrhythmias.^[51]^ There are reported cases of atrial arrhythmias, ventricular tachycardia, and syncopal episode secondary to ventricular tachycardia in patients of carcinoid syndrome.^[5,51]^ Further, there is also one reported case of carcinoid syndrome causing ventricular tachycardia in a canine subject.^[52]^

### 2.20. Myocardial metastases

Tumor cells can metastasize to the cardiac muscle leading to direct myocardial involvement and incidence of metastasis is 3.8%.^[53]^ The commonest site for metastases was the ventricles presenting as well circumscribed, homogenous mass and non-infiltrative on echocardiogram.^[54]^ Extent of metastases to heart reflects the degree of tumor burden, however, cardiac carcinoid metastases did not correlate with other classic valvular lesions of the carcinoid heart diseases.^[55,56]^ Cardiac carcinoid metastasis may be asymptomatic presenting as a solitary atrial mass, or it can cause ventricular outflow tract obstruction and these tumors are usually well-circumscribed and noninvasive, so excision may be beneficial.^[5]^

### 2.21. Pericardial involvement

Metastatic disease extending to pericardium is not very common.^[57]^ In our literature review, we found reported cases of chronic pericarditis and fibrosis and a case of acute pericarditis, secondary to CT.^[57,58]^ Also, there are 2 reported cases of constrictive carcinoid pericarditis without valve involvement.^[57,59]^ Together with this, a rare case of carcinoid pericardial metastases with massive effusion has been reported.^[60]^

## 3. Discussion

Carcinoid syndrome presents with diarrhea, cutaneous flushing, wheezing and asthma-like symptoms and pellagra-like skin lesions with hyperkeratosis and pigmentation.^[61]^ Diarrhoea is seen in carcinoid syndrome before of the tumoral secretion of 5HT.^[61]^ Circulating serotonin is exclusively derived from the GI tract and is an important component of normal gut function but excess serotonin, as secreted by the tumors of enterochromaffin cells, increases peristalsis, which results in reduced absorption of water and electrolytes, leading to diarrhea.^[61]^ Flushing occurs because of the vasoactive action (vasodilator) of 5HT on cutaneous blood vessels.^[62]^ Pellagra been reported in patients with carcinoid-syndrome because tryptophan is a precursor for both niacin and serotonin and the large increase in serotonin synthesis may lead to niacin depletion causing pellagra, though, Pellagra is not very common in carcinoid syndrome but Niacin deficiency is often documented, occurring 10 out 36 (28%) of patients in one study.^[63,64]^

Carcinoid-syndrome is the most frequent of the neuroendocrine tumors ectopic hormonal syndromes and is the second oldest, being described with its association with small bowel neuroendocrine tumors likely due to ectopic secretion of serotonin in 1954.^[63,64]^ Sad part of the disease is, it is very difficult to treat a patient of carcinoid syndrome and many cases are refractory to treatment.^[63,64]^ With advancement in genetics and molecular biology, Increasing insights pathogenesis of the disease have occurred, however, relatively few new treatments, or few controlled trials occurred until the last decade or more recently.^[64]^ Talking about the recent advances made in treatment strategies, release of tryptophan hydroxylase-1 inhibitor (telotristat), use of peptide radioreceptor therapy using radiolabeled somatostatin analogues which effective against both carcinoid syndrome and retarding growth of the tumor, use of ^131^MIBG (meta-iodobenzylguanidine), liver-directed therapies like radioembolization, chemo-embolization, and embolization, use of mega dose of somatostatin, tumorocidal and other procedures like surgical debulking of the tumor.^[64]^

## 4. Conclusion

Serotonin induced TGF-β activation via Gq mediated action seems to be the major pathway of fibrogenic activity in ICs, leading to carcinoid heart diseases. Serotonin induces transforming growth factor-beta1 mRNA in a concentration-dependent and time-dependent fashion, it binds to 5-HT_2A_ receptor which is Gq mediated and with binding of serotonin, α subunit of the receptor get deattached from β and γ subunit which stimulated phospholipase C, phospholipase C converts phospholipids into intositol triphosphide (IP3) and diacyl glycerol (DAG). DAG goes on to activate another enzyme, protein kinase C (PKC). The other intermediate produced, IP3, induces the release of calcium ions from the sarcoplasmic or endoplasmic reticulum. PKC leads to formation of NAD(P)H oxidase/reactive oxygen species, which in turn activates MEK, MEK activates ERK which finally acts as a transcription factor for TGF-beta1 mRNA.

Apart from that direct entry of 5HT into the cells due to downregulation of SERT, association of Renin–Angiotensin–Aldosterone system with 5HT induced fibrogenesis are few other interesting facts we found. Talking about the screening part, as already mentioned, higher level of 5HT in serum in patient of CT are destined to develop cardiovascular manifestations of CT. Hence, monitoring serum 5HT can be a good screening technique in patient of CT. Together with that increased urinary metabolite of serotonin (5-hydroxyindoleacetic acid) has also been in patient of CT. So, quantitative assay of 5-hydroxyindoleacetic acid in urine can be another alternative to screen the disease.

So far the treatment is concerned, targeting TGF-β and selective 5HT_2B_ receptor antagonist can be a potential way of management of carcinoid heart disease. Similarly, agents to upregulate the activity of SERT and Renin Angiotensin Aldosterone axis modulating agents can also be of help to these patients.

However, it is important to recognize the constraints of current research, such as limited sample sizes, publication bias, and the requirement for more thorough investigations. Clinicians must to adopt a comprehensive approach when addressing the management of carcinoid heart disease, taking into account the unique requirements of each patient and thoroughly evaluating the potential advantages and disadvantages associated with each therapeutic option. In order to provide evidence-based guidelines for the management of this complicated and problematic condition, it is recommended that future research endeavors prioritize the implementation of bigger, well-designed studies. This study serves as an excellent source for clinicians who are seeking guidance in managing the complexities of carcinoid heart diseases in order to deliver optimal treatment for persons impacted by this condition.

## Author contributions

**Conceptualization:** Sajjad Ahmed Khan.

**Data curation:** Sajjad Ahmed Khan, Anish Luitel.

**Methodology:** Rahul Kumar Chaudhary.

**Resources:** Dikshya Devkota.

**Software:** Dikshya Devkota.

**Supervision:** Surya Bahadur Parajuli, Madhab Bista, Asmita Rayamajhi.

**Writing – original draft:** Sajjad Ahmed Khan.

**Writing – review & editing:** Surya Bahadur Parajuli.
